# Differences Between Total Ankle Replacement and Ankle Arthrodesis in Post-operative Complications and Reoperations at 30 Days and One Year

**DOI:** 10.7759/cureus.28703

**Published:** 2022-09-02

**Authors:** Senthil Sambandam, Philip Serbin, Dietrich Riepen, Vikram A Aggarwal, Varatharaj Mounasamy, Dane Wukich

**Affiliations:** 1 Orthopedics, University of Texas Southwestern Medical Center, Dallas, USA

**Keywords:** database review, reoperations, complications, total ankle replacement, ankle arthrodesis

## Abstract

Purpose

Total ankle replacement (TAR) and ankle arthrodesis (AA) are two commonly performed procedures for end-stage arthritis of the ankle joint. The aim of this study was to analyze the differences in the rates of complications and reoperations at both 30 days and one year within a matched sample of TAR and AA patients from a large database population.

Methods

A commercially available patient database record, known as the PearlDiver database (www.pearldiverinc.com, Colorado Springs, CO, USA), was used for this study. Patients undergoing TAR and AA were identified using Current Procedural Terminology (CPT) codes. After matching both TAR and AA groups for confounding variables, such as diabetes, smoking, obesity, and comorbidities scores, the differences in the rates of complications at 30 days and one year and the rate of reoperation at one year were evaluated in both groups.

Results

After matching for confounding variables, there were 1287 patients in each group. There was no significant difference in the male/female ratio. Within each group, 430 patients were diabetic, 102 patients smoked, and 543 patients were obese. The rate of surgical site infection (SSI) and wound dehiscence were higher at 30 days in the AA group. About 63.45% of complications happened after 30 days. The AA group showed a higher rate of SSI, wound dehiscence, mechanical complications, and pneumonia at one year. The rate of reoperation was also higher in the AA group at one year.

Conclusion

Ankle arthrodesis is associated with a higher rate of local and systemic complications at 30 days and one year, along with a higher reoperation rate at one year, when compared to total ankle replacement. Most complications happened after 30 days, suggesting that studies reporting complications within 30 days following AA and TAR may underestimate the true rates of complications.

## Introduction

Ankle arthritis is a commonly encountered clinical condition in foot and ankle practice. Total ankle replacement (TAR) and ankle arthrodesis (AA) are two commonly performed procedures for end-stage arthritis of the ankle. AA has been the gold standard for several decades, and TAR has gained in popularity over the past two decades [1,2]. TAR has the theoretical advantage of maintaining and restoring mobility and improving gait, thereby reducing stress on the subtalar and transverse tarsal joints. This stress reduction then reduces the risk of adjacent joint arthritis. Various aspects of TAR and AA, including gait, kinematics, patient expectation, and satisfaction, have been extensively studied by different authors [3-5]. However, there is a significant lack of consensus in the literature regarding the overall risks of complications and reoperations pertaining to TAR and AA. For example, Maffulli et al. [6] in their review of the literature concluded that TAR could not be routinely recommended for the management of ankle arthritis. However, Li et al. [7] concluded that there is no statistically significant difference between TAR and AA with regard to clinical outcome, patient satisfaction, complications, and survival. Therefore, the aim of this study is to compare the rates of complications and reoperations at both 30 days and one year among patients who have undergone TAR and AA.

## Materials and methods

In this retrospective study, we used the PearlDiver patient record database (www.pearldiverinc.com, Colorado Springs, CO, USA). PearlDiver is a commercially available database from Medicare and several different private insurers with more than 30 million patient records. The data in Pearl Diver has been extensively used in orthopedic research and is compliant with the Health Insurance Portability and Affordability Act (HIPPA). For the purposes of this study, we used the Humana subset (MSOrtho30) of the database and included all patients treated between 2010 and 2019. All patients undergoing TAR and AA were identified using Current Procedural Terminology (CPT) codes 27702 for TAR and 27870 and 29899 for AA.

The AA group was matched to the TAR group with respect to age, gender, presence of diabetes, smoking history, obesity, Charlson comorbidity index (CCI), and Elixhauser comorbidity index (ECI). CCI comprises 19 comorbidities, including diabetes, congestive heart failure, peripheral vascular disease, cerebrovascular disease, liver disease, renal disease, neurological diseases, and hematological diseases. ECI encompasses 31 comorbidities and includes obesity, hypertension, and psychiatric disorders that are not included in CCI. A weighted score was assigned to each comorbidity, and an overall score was calculated by summing the weighted values. Higher values are associated with a higher disease burden and a higher 10-year mortality risk. This matching yielded a total of 1287 patients in each group. Complications, including acute kidney injury (AKI), cardiac arrest, cerebrovascular accident (CVA), deep vein thrombosis (DVT), hematoma formation, myocardial infarction (MI), pulmonary embolism (PE), nerve injury, pneumonia, transfusion, wound complications, mechanical complications, wound dehiscence, and surgical site infection (SSI), in each group were identified using International Classification of Diseases, Ninth Revision (ICD-9) codes. We then compared the differences in the rates of complications and reoperations between the two groups at 30 days and one year.

## Results

Baseline characteristics of the matched sample

There were 657 females and 630 males in the two matched groups (Table 1). Five hundred and forty-three patients were documented to have obesity, 430 patients had diabetes, and 102 patients smoked. Nine hundred and sixty-four patients had a CCI of 0, 269 patients had a CCI of 2 or 3, and 46 patients had a CCI above 3. Nearly one-third of the patients (447 patients) were in the 55 to 64 age group.

**Table 1:** Characteristics of the matched cohort. CCI: Charlson comorbidity index.

Total matched cohort	1287
Female	657 (51.04%)
Male	630 (48.96%)
Obesity, Yes	543 (42.19%)
Obesity, No	744 (57.81%)
Diabetes, Yes	430 (33.41%)
Diabetes, No	857 (66.6%)
Smoking, Yes	102 (7.9%)
Smoking, No	1185 (92.1%)
CCI 0	964 (74.9%)
CCI 1 or 2	269 (20.9%)
CCI 3 or 4	46 (3.5%)
Age 20-24	21 (1.6%)
Age 25-29	30 (2.3%)
Age 30-34	37 (2.8%)
Age 35-39	60 (4.6%)
Age 40-44	86 (6.6%)
Age 45-49	136 (10.56%)
Age 50-54	213 (16.55%)
Age 55-59	234 (18.18%)
Age 60-64	175 (13.59%)
Age 70-74	194 (15.07%)
Age 75-79	81 (6.2%)

Complications

Complications like cardiac arrest, cerebrovascular accident, DVT, hematoma formation, myocardial infarction, pulmonary embolism, nerve injury, and need for transfusion were rare and reported in less than 11 patients in both groups at 30 days and one year. The PearlDiver database does not report the exact number of events if the number is less than 11 for patient’s privacy reasons.

Local complications were more prevalent both in patients undergoing TAR and AA when compared to systemic complications. The most common local complication noted in both groups was SSI. The second most common local complication was wound dehiscence in the TAR group and mechanical complications in the AA group. The two most common systemic complications noted in this study were pneumonia, followed by AKI (Table 2).

**Table 2:** Complications at 30 days and one year AKI: acute kidney injury, SSI: surgical site infection.

	AA	TAR	Odds ratio (P-value)
AKI at one year	28	18	1.56 (p=0.14)
AKI at 30 days	<11	<11	NA
Wound dehiscence one year	49	56	0.87 (p=0.48)
Wound dehiscence 30 days	13	<11	NA
Mechanical complications one year	79	30	2.74 (p=0.0001)
Mechanical complications 30 days	20	<11	NA
Pneumonia at one year	35	20	1.77 (p=0.04)
Pneumonia at 30 days	13	<11	NA
SSI at one year	92	52	1.82 (p=0.0007)
SSI at 30 minutes	38	12	3.23 (p=0.0004)
Transfusion at one year	15	11	1.36 (p=0.43)
Transfusion at 30 days	<11	<11	NA
Wound complications at one year	55	51	1.08 (p=0.69)
Wound complications at 30 days	23	12	1.93 (p=0.06)

There was no significant difference between those patients who underwent TAR or AA with regard to medical complications, such as AKI or the need for transfusion. Patients who underwent AA had a significantly increased rate of experiencing pneumonia during the first postoperative year compared to patients who underwent TAR (OR=1.77, P=0.04).

Thirty-day complication rate

Surgical site infection was significantly higher in patients who underwent AA compared to patients who underwent TAR. There was also a strong trend toward higher wound complication rates in patients who had AA compared to TAR (P=0.06).

One-year complication rate

There was no significant difference in the wound dehiscence or wound complication rate at one year between the two groups. Surgical site infections were significantly increased in the AA group at one year compared to the TAR group (OR=1.82, p=0.0007) with more than 50% of systemic and local complications occurring after 30 days. Therefore, studies reporting on 30-day complication rates may be under-reporting the complication rate after AA and TAR.

Reoperation after total ankle fusion and replacement

Non-revision operations were performed for infection, seroma, hematoma, and wound dehiscence. Revision operations were revision arthroplasty for the TAR group and non-union repair for AA. The rate of reoperation for wound complications, such as hematoma, seroma, or wound dehiscence, was minimal and often needed in less than 11 patients in each group (Table 3). The reoperation rate for infection was significantly higher in patients who underwent AA versus TAR (OR=1.46, p=0.03), with infection being the most common reason for reoperation in both groups. There was a trend toward significance in the overall total reoperation rate in patients who underwent AA compared to TAR. While this difference did not reach statistical significance with the numbers involved, we feel that it is clinically meaningful information (OR=1.30, p=0.08).

**Table 3:** Reoperations at one year. AA: ankle arthrodesis, TAR: total ankle replacement.

	AA	TAR	Odds ratio (P-value)
Reoperation for infection	76 (5.9%)	53 (4.1%)	1.46 (p=0.03)
Reoperation for dehiscence	<11	13	NA
Reoperation hematoma	12	<11	NA
Reoperation for seroma	<11	0	NA
Reoperation for non-union	13	0	NA
Revision ankle replacement	<11	13	NA
Total reoperation	101(7.8%)	79 (6.1%)	1.30 (p=0.08)

## Discussion

This large database study demonstrates that the rate of SSI and the rate of reoperation for infection are higher in patients who undergo AA compared to TAR. Most complications following AA and TAR occur after 30 days. Hence, studies reporting only on the 30-day complicated rate most likely under-report the actual rate of complications.

Probasco et al. [8] analyzed the difference in the complication rate at 30 days between AA and TAR and found no difference between local and systemic complications. Jiang et al. [9] also analyzed the differences in the rates of in-hospital medical complications between the AA and TAR groups and found similar rates of pneumonia, DVT, PE, CVA, MI, and mortality. Odum et al. [10] looked at the rate of major and minor complications during in-hospital stay after TAR and AA, including systemic and local complications in both groups. Overall, the AA group was associated with a higher rate of major complications compared to the TAR group. Our study shows there was no difference in the rate of systemic complications at 30 days, however, there was a significantly higher rate of SSI in the AA and this difference was maintained at one-year follow-up as well. In contrast to Probasco’s, Odum’s, and Jiang’s studies looking at only 30-day complication rates, we report on complications up to one year postoperatively. Although we did not identify any difference in systemic complications at 30 days, AA patients experienced significantly higher rates of pneumonia when the follow-up period was extended to one year. We also noted that for several complications like AKI, wound dehiscence, and other wound complications, more than 50% of cases happened after 30 days, thereby highlighting the importance of a one-year follow-up versus a 30-day follow-up.

In his pooled sample analysis, Lawton reported the overall long-term complications rates with an average follow-up of more than four years [11]. This study noted an overall complication rate of 19.9% for the TAR group and 26.9% for the AA group. Similar to our study, they reported that the rate of infection and wound complications was higher in the AA group.

The overall rate of reoperation in our study was 7.8% for the AA group and around 6.9% for the TAR group. While this did not reach statistical significance, we feel that it is clinically meaningful. Infection was the most common reason for reoperation in our study. Norvell et al. [12] and Lawton et al. [11] reported the rate of major reoperations and minor reoperations at 24 months. They noticed an increased rate of minor reoperations after AA but no difference in the rate of major reoperations, which is similar to the findings of our study. In contrast, SooHoo et al. [13], in a California hospital discharge database study, reported a higher rate of major complications after TAR at one year and five years. Younger et al. [14] in their prospective cohort study reported a higher reoperation rate for TAR (30%) versus 14% for AA. A potential limitation of these studies is the failure to match the AA and TAR cohorts with respect to comorbidities. SooHoo et al. [13] showed a significant difference in the baseline demographic variables like race, preoperative diagnosis, and incidence of complicated diabetes, which potentially introduces selection bias.

Limitations

Our study has limitations inherent to most large database studies. The grouping of TAR and AA patients was based on CPT codes and, hence, we were not able to analyze the impact of the primary diagnosis on the rates of complications and reoperations. Also, patient-level data is not available, leading to a loss of granularity. Finally, the accuracy of the data is highly dependent on the accuracy of coding.

## Conclusions

Our study showed most local and systemic complications are noted to occur after 30 days. Consequently, studies that report on the 30-day complication rate may underestimate the true complication rate. AA had a higher complication rate compared to TAR in this cohort of matched patients. In particular, the AA group had a higher rate of surgical infection and a higher rate of reoperation for infection compared to the TAR group.
